# Association between depression and brain tumor: a systematic review and meta-analysis

**DOI:** 10.18632/oncotarget.19843

**Published:** 2017-08-03

**Authors:** Jing Huang, Chao Zeng, Juxiong Xiao, Danwei Zhao, Hui Tang, Haishan Wu, Jindong Chen

**Affiliations:** ^1^ Department of Psychiatry, The Second Xiangya Hospital, Central South University, Changsha, Hunan, China; ^2^ Mental Health Institute of The Second Xiangya Hospital, Central South University, Chinese National Clinical Research Center on Mental Disorders (Xiangya), Chinese National Technology Institute on Mental Disorders, Hunan Key Laboratory of Psychiatry and Mental Health, Changsha, Hunan, China; ^3^ Department of Thoracic Surgery, Second Xiangya Hospital of Central South University, Changsha, Hunan, China; ^4^ Department of Urology, Xiangya Hospital, Central South University, Changsha, Hunan, China; ^5^ Xiangya Medical School, Central South University, Changsha, Hunan, China

**Keywords:** brain tumor, depression, depressive disorder, depressive symptoms, meta-analysis

## Abstract

**Background:**

Patients with brain tumor are in risk of depression or depressive symptoms, but the estimated prevalence varies between studies. The aim of this study is to get a proper summarized estimate of depression prevalence in brain tumor patients.

**Methods:**

Literature search on Pubmed, PsycINFO, and Cochrane library from January 1981 through October 2016. The prevalence of depression or depressive symptoms in brain tumor patients was estimated by screening scales and analyzed using stratified meta-analysis and subgroup analysis. The prevalence of depression level or symptoms during the follow-up periods was detected by secondary analysis.

**Results:**

Among the 37 studies included in this meta-analysis, 25 used a cross-sectional design and 12 used longitudinal study. The pooled prevalence was 21.7% (971/4518 individuals, 95 % confidence interval (CI) 18.2%–25.2%) for overall sample. Lower prevalence was detected in studies with sample size ≥100 than <100, lower grade tumor than high grade tumor, studies using clinician-rated depression scales than self-rated or non-depression-specific ones, and in patients from UK, Germany and Italy than USA. After analyzing 6 longitudinal studies, prevalence of depression remained no change in the follow-up periods. No significant differences were observed between study designs and tumor types.

**Conclusions:**

The estimated prevalence of depression or depressive symptoms among brain tumor patients was 21.7%, affected by depression assessment type, sample size, tumor grade and country. Diagnosis and treatment of co-morbid depression in brain tumor patients need to be addressed in future studies for better life quality and oncology management.

## INTRODUCTION

Depression is a severe mental health disorder developed under different circumstances, formally diagnosed by DSM-IV or DSM-V (Diagnostic and Statistical Manual of Mental Disorders 4^th^ edition or 5^th^ edition) [1, 2]. Depressive symptoms, such as fatigue, loss of interest, decreased energy, feelings of guilt, worthlessness could be main manifestations of depressive disorder or other psychological diseases [1, 2]. Depression or depressive symptoms among brain tumor patients have been reported by distinct diagnostic clinical interviews with distinct criteria and thresholds [3, 4], which have been linked to the adverse course of the disease, a worsened life quality and even higher rates of mortality [4–8]. However, estimates of the prevalence of depression or depressive symptoms varied greatly, ranging from 2.8% to 95% [9, 10]. Different screening and diagnostic scales were employed to evaluate depression prevalence in brain tumor patients with different age or sex, education level, countries, brain tumor type and grade, thus leading to various findings about the estimated depression prevalence [11–14].

The adverse impacts of depression or depressive symptoms among patients with brain tumor, the various risk factors and the variations between assessment tools, have made it an urgent task to obtain an accurate and reliable depression prevalence in brain tumor patients. The aim of our study is to acquire a proper summary estimate of the depression prevalence and to discuss the reasonable and suitable depression assessment instruments in the clinical setting. Therefore, we conducted a systematic review and meta-analysis from 37 observational studies, to get a summary prevalence of depression among brain tumor patients and help to develop a better identification, prevention and treatment of the depression co-morbidity and original tumor.

## RESULTS

### Selection of studies and study characteristics

**Figure 1:**
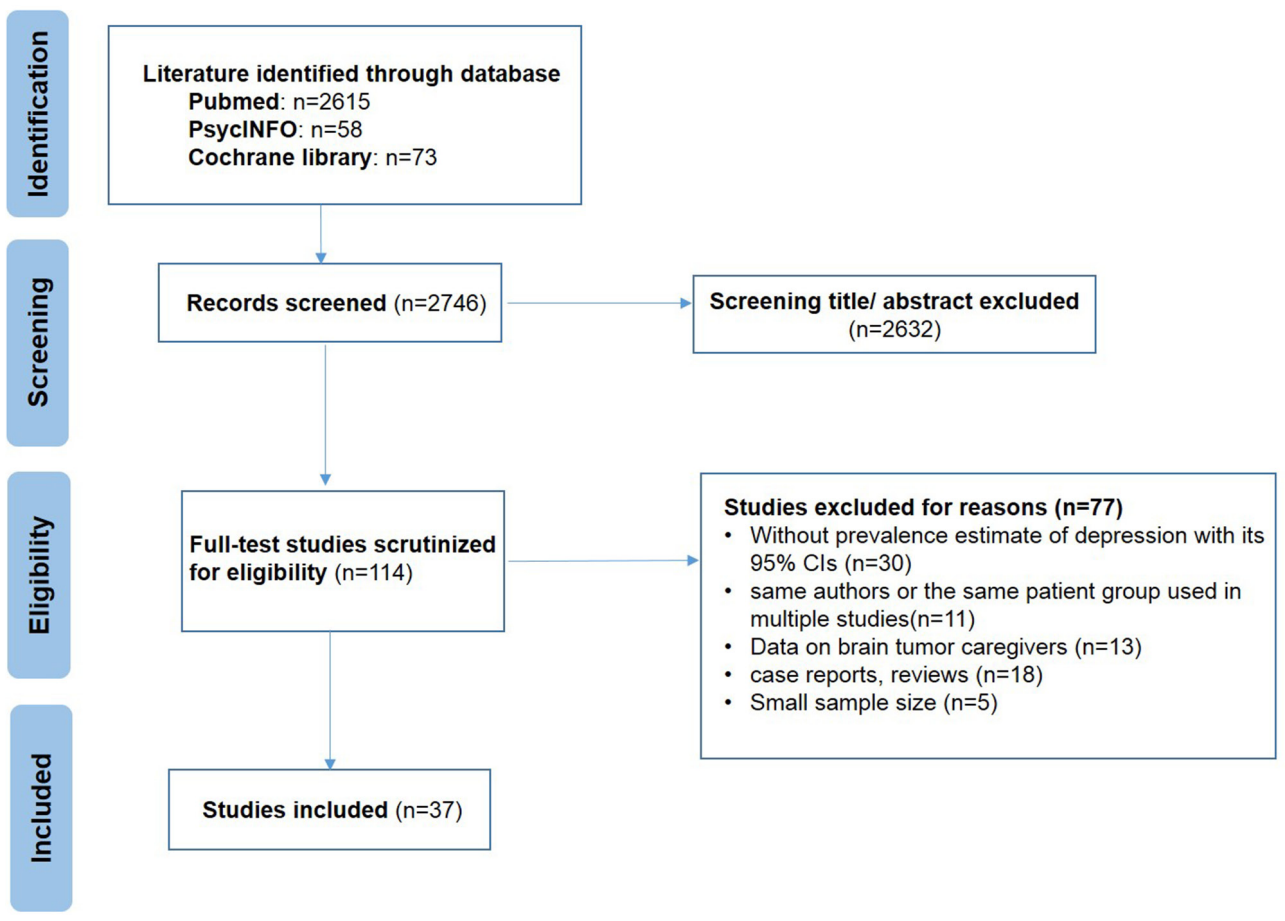
Meta-analysis flowchart for identifying studies on the prevalence of depression among brain tumor patients

The initial search strategy identified 2746 potentially articles: 2615 from PUBMED, 73 from Cochrane library, and 58 from PsycINFO. Figure 1 presented details of the studies included in the meta-analysis. After screening the titles and abstracts according to the selection criteria, we excluded 2622 studies. We also identified additional studies by reference scanning and previous meta-analysis or reviews. Overall, we got a total of 37 eligible studies for further analysis.

### Main associations of depression with brain tumor

These studies provided a total sample of 4518 patients (median sample size = 122 patients, range = 22–573 patients) including 25 cross-sectional [4, 5, 12, 14, 19, 22–24, 33–49] studies, 12 longitudinal studies [6, 7, 13, 20, 21, 50–56]. No randomized controlled trial was eligible. All 37 studies are prospective research. The average percentage of men in the total sample was 51.3%. 17 studies assessed for depression or depressive symptoms using Hospital Anxiety and Depression Scale (HADS-D) [4, 7, 13, 14, 21, 33, 35, 39–43, 45, 46, 49–51], 6 used Beck Depression Inventory (BDI) [5, 6, 23, 44, 52, 54, 57], 2 used the Zung Self-Rating Depression Scale (Zung SDS) [51, 58], 2 used Diagnostic and Statistical Manual of Mental Disorders, 4th. Edition (DSM-IV) [12, 47], 10 used other methods [19, 20, 22, 24, 34, 36, 37, 48, 53, 56]. The diagnostic criteria used by the studies were summarized in Table 1. When evaluated by the modified Newcastle-Ottawa scale, out of 5 possible points, 0 studies received 5 points, 6 received 4 points, 18 received 3 points, 9 received 2 points, 4 received 1 point, and 0 received 0 points (detailed criteria were presented in the Supplementary 2).

**Table 1:** Characteristics of studies included in this systematic review and meta-analysis

First author	Year	Country	Study design	Recuitment	Patients, n	Male patients, n (%)	Age, y, mean	Brain tumor type	WHO low-grade, n	WHO high-grade, n	Surgery,%	Education≥high school,%	Married, %	Previous psychiatric illness,%	White,%	Depression scale
Hickmann	2016	Switzerland	Longitudinal	Prospective	83	43.4	51.9	multiple	51	31	98.8	30	NR	NR	NR	BDI
Jenkins	2015	Australia	cross-sectional	Prospective	33	NR	45.75	multiple	0	30	NR	NR	NR	NR	NR	HADS-D
WELLISCH	2002	USA	cross-sectional	Prospective	89	55	43.2	multiple	NR	39	73	67.1	61.8	15.8	NR	DSM-IV
Arnold	2008	USA	cross-sectional	Prospective	363	58	43.7	multiple	219	144	NR	83	76	5	95	PHQ-9
Anderson	1999	UK	cross-sectional	Prospective	40	60	44	glioma	24	16	83	NR	70	NR	NR	HDS
Davies	1996	UK	Longitudinal	Prospective	75	69	NR	multiple	0	75	NR	NR	78	NR	93	open ended interviews
Pringle	1999	UK	cross-sectional	Prospective	109	56.88	NR	multiple	53	32	93	NR	NR	NR	NR	HADS-D
Litofsky	2004	USA	Longitudinal	Prospective	573	58	55	glioma	0	598	81.4	NR	80	NR	92.5	SF-36
Pelletier	2002	Canada	cross-sectional	Prospective	58	51.67	41.1	multiple	18	34	90	95	66.6	NR	NR	BDI-II
Edelstein	2015	USA	cross-sectional	Prospective	73	60.3	NR	glioma	0	73	NR	NR	83.6	NR	NR	CES-D
Wenz	2015	Germany	cross-sectional	Prospective	58	72.2	62.6	meningioma	58	0	77.9	NR	NR	20.83	NR	BCS
Piil	2015	Denmark	Longitudinal	Prospective	28	63.3	60	glioma	0	30	76.67	NR	80	NR	NR	HADS-D
Rahman	2015	Australia	cross-sectional	Prospective	81	58	NR	multiple	30	51	100	58	NR	NR	NR	HADS-D
Leistner	2015	Germany	cross-sectional	Prospective	247	37	53.25	pituitary adenoma	0	0	66.7	NR	NR	NR	NR	BDI
Lucchiari	2014	Italy	cross-sectional	Prospective	73	66	48.9	glioma	0	73	NR	17.8	NR	NR	NR	HADS-D
Janda	2007	Australia	cross-sectional	Prospective	75	45.9	74.6	multiple	31	44	NR	70.2	62.2	NR	NR	HADS-D
Vossen	2014	Netherlands	cross-sectional	Prospective	136	22	59.1	meningioma	134	2	71	40	NR	NR	NR	HADS-D
ANGELO	2008	Italy	Longitudinal	Prospective	72	43.1	NR	multiple	22	10	NR	13.9	79.17	NR	NR	Zung SDS
Bunevicius	2012	Lithuania	Longitudinal	Prospective	226	31	55.6	multiple	3	65	NR	NR	NR	7.1	NR	HADS-D
Andrewes	2013	Australia	cross-sectional	Prospective	32	43.8	52	multiple	0	29	NR	43.8	NR	NR	NR	HADS-D
Goebel	2012	Germany	Longitudinal	Prospective	76	33	54.42	meningioma	52	24	100	NR	84	11.8	NR	HADS-D
Keeling	2012	UK	cross-sectional	Prospective	74	46	38.3	multiple	64	0	68.66	NR	NR	NR	NR	HADS-D
Goebel	2012	Germany	cross-sectional	Prospective	172	48.8	52.4	multiple	93	78	NR	NR	NR	NR	NR	HADS-D
Santini	2012	Italy	Longitudinal	Prospective	22	45	NR	multiple	14	8	100	NR	NR	NR	NR	BDI
Mainio	2006	Finland	Longitudinal	Prospective	77	38.6	NR	glioma	16	15	NR	NR	NR	NR	NR	BDI
Kilbride	2007	UK	Longitudinal	Prospective	51	54.9	55	multiple	3	42	100	NR	NR	NR	NR	HADS-D
Rooney	2011	UK	Longitudinal	Prospective	155	57.4	NR	glioma	22	133	74.8	NR	80	18.06	NR	DSM-IV
Goebel	2011	Germany	cross-sectional	Prospective	180	48.3	52.7	multiple	NR	78	NR	NR	75.6	NR	NR	HADS-D
Armstrong	2002	USA	Longitudinal	Prospective	57	NR	40.77	glioma	57	0	67	NR	NR	NR	NR	BDI
Brown	2006	USA	cross-sectional	Prospective	185	65.5	NR	glioma	0	185	83.5	NR	NR	NR	NR	POMS-SF
CHANG	2003	USA	cross-sectional	Prospective	499	55.7	NR	glioma	0	499	91.8	NR	NR	NR	NR	Physician report
Giovagnoli	1996	Italy	cross-sectional	Prospective	125	101	60	multiple	NR	11	90	NR	NR	70	NR	NR
Grant	1994	UK	cross-sectional	Prospective	48	NR	NR	glioma	NR	NR	NR	NR	NR	NR	NR	HADS-D
Kaplan	2000	USA	cross-sectional	Prospective	33	NR	33	multiple	0	33	NR	NR	75.8	NR	NR	BDI
McGovern	2003	USA	cross-sectional	Prospective	33	NR	NR	multiple	0	33	NR	NR	NR	NR	NR	Inpatient notes
Rooney	2009	UK	cross-sectional	Prospective	100	55	NR	glioma	NR	NR	NR	NR	NR	NR	NR	GP records
Goebel	2010	Germany	cross-sectional	Prospective	150	43.3	53.15	multiple	73	77	NR	NR	64.3	NR	NR	HADS-D

BDI, Beck Depression Inventory; HADS-D, Depression Subscale of Hospital Anxiety and Depression Scale; DSM-IV, Diagnostic and Statistical Manual of Mental Disorders, 4th. Edition; PHQ-9; Patient Health Questionnaire–9; HDS, Hamilton Rating Scale for Depression; SF-36, 36-Item Short Form Health Survey; BDI-II, Beck Depression Inventory-II; CES-D, Center for Epidemiologic Studies-Depression Scale; BCS, Brief Cope Scale; Zung SDS, Zung Self-rating Depression Scale; POMS-SF, Profiles of Mood States Short Form; GP, General Practitioner (family physician); SF-36, 36-Item Short Form Health Survey; NR, not applicable.

**Figure 2:**
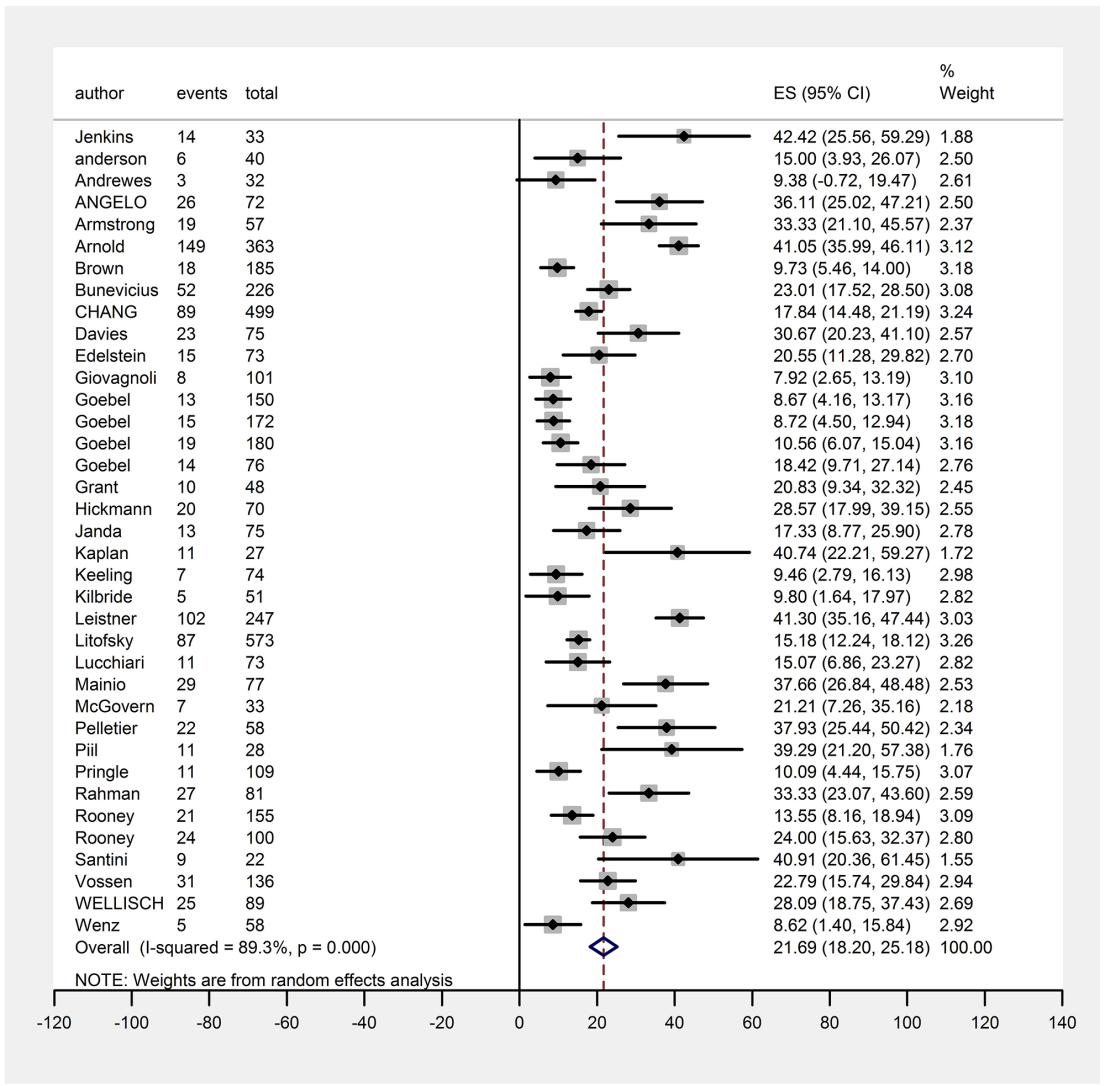
Forest plot for random-effects meta-analysis showing pooled prevalence of depression in overall sample

First, we compared depression prevalence in the overall sample. Random-effects meta-analysis was performed. And the results showed that the pooled prevalence of depression disorder in brain tumor patients was 21.7% (971/4518 individuals, 95 % confidence interval (CI) 18.2%–25.2%) in the overall sample (Figure 2). Significant evidence of between-study heterogeneity was observed in the meta-analysis (I^2^ = 89.3%, P <0.01). The results of sensitivity analysis were not influenced by an individual study by more than 1% (Supplementary 3).

### Subgroup analysis

We next compared the prevalence of depression or depressive symptoms depending on different demographic groups, depression scales and other characteristics by a series of sub-group analyses (Table 2 and Supplementary 4). No significant differences were observed between studies stratified by cross-sectional vs longitudinal studies (696/3131, 20.7% [95% CI, 16.2% to 25.2%] vs 275/1387, 24.0% [95% CI, 18.1% to 29.8%]; test for subgroup differences, Q =0.58, P =0.45), tumor types investigated including glioma only vs multiple tumor types such as glioma, meningioma, pituitary adenoma (340/1908, 19.6% [95% CI, 15.6% to 23.5%] vs (631/2610, 22.5% [95% CI, 17.4% to 27.6%]; Q = 2.89, P = 0.09). Heterogeneity was partly explained by large sample size (sample ≥100) vs small sample size (sample <100) (668/3273, 19.1% [95% CI, 13.9% to 24.3%] vs 303/1245, 23.8% [95% CI, 19.2% to 28.4%]); Q = 9.18, P <0.01), countries patients recruited (studies in the United States vs UK vs Germany vs Italy vs elsewhere (420/1899, 24.3% [95% CI, 16.9% to 31.7%] vs 119/831, 14.8% [95% CI, 10.1% to 19.6%] vs 132/510, 16.6% [95% CI, 4.2% to 29.1%] vs 68/344, 21.7% [95% CI, 10.9% to 32.4%] vs 232/934, 27.7% [95% CI, 20.4% to 35.1%]; Q = 33.01, P ≤0.01)). Significant prevalence difference between high grade glioma (WHO I and II) vs low grade glioma (WHO III and IV) was also detected (48/418, 19.5% [95% CI, 13.9% to 25.1%] vs 180/1133, 15.4% [95% CI, 6.4% to 24.4%]; Q = 16.57, P <0.01) (Supplementary 5).

**Table 2:** Meta-analyses of the prevalence of depression or depressive symptoms among brain tumor patients stratified by study-level characteristics

	No. of studies	No of patients with depression	Total number of patients	Prevalence of depression, %(95%Cl)	P for subgroup differences
Study Design					
Longitudinal	12	275	1387	24.0 (18.1-29.8)	0.45
cross-sectional	25	696	3131	20.7 (16.2-25.2)	
Country					
USA	9	420	1899	24.3 (16.9-31.7)	<0.01
UK	7	119	831	14.8 (10.1-19.6)	
Germany	6	132	510	16.6 (4.2-29.1)	
Italy	5	68	344	21.7 (10.9-32.4)	
Others	10	232	934	27.7 (20.4-35.1)	
Sample size					
≥100	15	668	3273	19.1 (13.9-24.3)	<0.01
<100	22	303	1245	23.8 (19.2-28.4)	
Tumor type					
glioma	12	340	1908	19.6 (15.6-23.5)	0.09
multiple	25	631	2610	22.5 (17.4-276)	
Type of depression assessment					
clinician-rated	6	172	916	19.1 (14.9-23.2)	0.018
self-rated	27	639	2711	20.6 (17.2-23.1)	
non-depression scales	4	133	891	14.8 (8.5-21.00)	

When we stratified studies by depression scales, high heterogeneity was detected (Q=273.83, P ≤0.01). Then we divided all the depression scales used by these studies into clinician-rated scales, self-rated scales and non-depression-specific scales, based on the type of depression assessment. Clinician-rated scales included DSM-IV, Hamilton Rating Scale for Depression (HDS) ≥17 [59], General Practitioner (GP) records [56], Inpatient notes [24] and Physical reports [36]. And self-rated scales included HADS-D with a cut-off ≥11 [60], and Patient Health Questionnaire–9 (PHQ-9) ≥10 [61, 62], BDI ≥10 [63], Beck depression inventory-II (BDI-II) ≥14 [64], Center for Epidemiologic Studies-Depression Scale (CES-D) ≥16 [65], HADS-D ≥8 [60], Zung SDS ≥41 [66]. Other studies which use non-depression-specific diagnostic methods were grouped as non-depression-specific scales, consist of Profiles of Mood States Short Form (POMS-SF) ≤50 [67], 36-Item Short Form Health Survey (SF-36) ≤60 [68], open ended interviews, as well as Brief Cope Scale (BCS). DSM-IV, as a clinician-rated scales, has obtained a status as the international standard for Major Depressive Disorder [2]. And HDS, GP records, inpatient notes and physical reports are physician-based depression symptoms rating in clinical practice. Self-rated depression scales, which are also widely applied in clinical setting, are considered as good screening tools for depressive disorder or symptoms. Non-depression-specific scales often recognize distressing emotional symptoms not restricted to depressive symptoms [69].

The high heterogeneity between studies could partly be explained by type of depression assessment (clinician-rated scales vs self-rated scales vs non-depression-specific scales (172/916, 19.1% [95% CI, 14.9% to 23.2%] vs (666/2711, 20.6% [95% CI, 17.2% to 23.1%] vs (133/891, 14.8% [95% CI, 8.5% to 21.0%]; Q = 14.96, P < 0.01)) (Supplementary 3E). There were no significant differences between studies in which estimates was made by clinician-rated scales (Q = 2.57, P = 0.63), suggesting that variation between clinical rated tools did not explain the heterogeneity in the symptom prevalence estimates. Conversely, there were significant differences between estimates using self-rated scales (Q = 16.35, P <0.01) and non-depression scales (Q = 202.44, P <0.01). These results indicated that in the clinical setting, physician based assessing tools are more stable and consistent for depression diagnosis.

### Secondary analysis

Of the 12 longitudinal studies, we detected prevalence of depression or depressive symptoms at different time points to figure out whether there was an increased prevalence with increasing calendar year or in further analysis. Patients after diagnosis at baseline were involved in follow-up studies. Follow-up time points varied across studies, from 3 months to 12 months. 6 studies were excluded because they are in lack of available raw data on prevalence of depression or their main focus is not on the outcome and effect of depression or depressive symptoms [39, 50, 52, 53, 55, 70]. After analyzing the remaining 6 longitudinal studies [6, 7, 20, 40, 51, 54], brain tumor patients presented with a slightly higher prevalence of depression in the follow-up period (Relative Increase Ratio:1.35, 95% CI(1.04, 1.76)) (P = 0.025) (Table 3). Sensitivity analysis for the secondary analysis revealed that Angelo’s study has substantial influence on the final result [51]. After moving out this study, the result showed that prevalence of depression remained no change in further analysis. (Relative Increase Ratio: 1.20, 95% CI(0.91, 1.59)) (P = 0.204).

**Table 3:** Secondary analysis of 6 longitudinal studies reporting prevalence estimates with increasing calendar year in further analysis

				Baseline			Follow-up			Comparison
First author	Year	Depression scale	Follow-up	No of patients with depression	Total number of patients	Prevalence of depression,%(95%Cl)	No of patients with depression	Total number of patients	Prevalence of depression,%(95%Cl)	Relative increase ratio,%(95%Cl)
Hickmann	2016	BDI ≥10	3 mo	19	70	27.1(16.7, 37.6)	20	70	28.6(18.0,39.2)	1.05 (0.52,2.14)
Litofsky	2004	SF-36 ≤60	6 mo	87	573	15.2(12.2,18.1)	42	193	21.8(15.9,27.6)	1.43 (0.96,2.14)
Piil	2015	HADS-D ≥11	6 mo	11	28	39.3(21.2,57.4)	5	26	19.2(4.0,34.4)	0.49 (0.15,1.60)
ANGELO	2008	Zung SDS ≥41	6 mo	7	72	9.7(2.9,16.6)	26	72	36.1(25.0,47.2)	3.71 (1.52,9.10)
Goebel	2012	HADS-D ≥11	6 mo	9	76	11.8(4.6,19.1)	14	76	18.4(9.7,27.1)	1.56 (0.64,3.81)
Mainio	2006	BDI ≥10	3 mo	27	77	35.1(24.4,45.7)	29	81	35.8(25.4,46.2)	1.02 (0.55, 1.88)

### Publication bias

**Figure 3:**
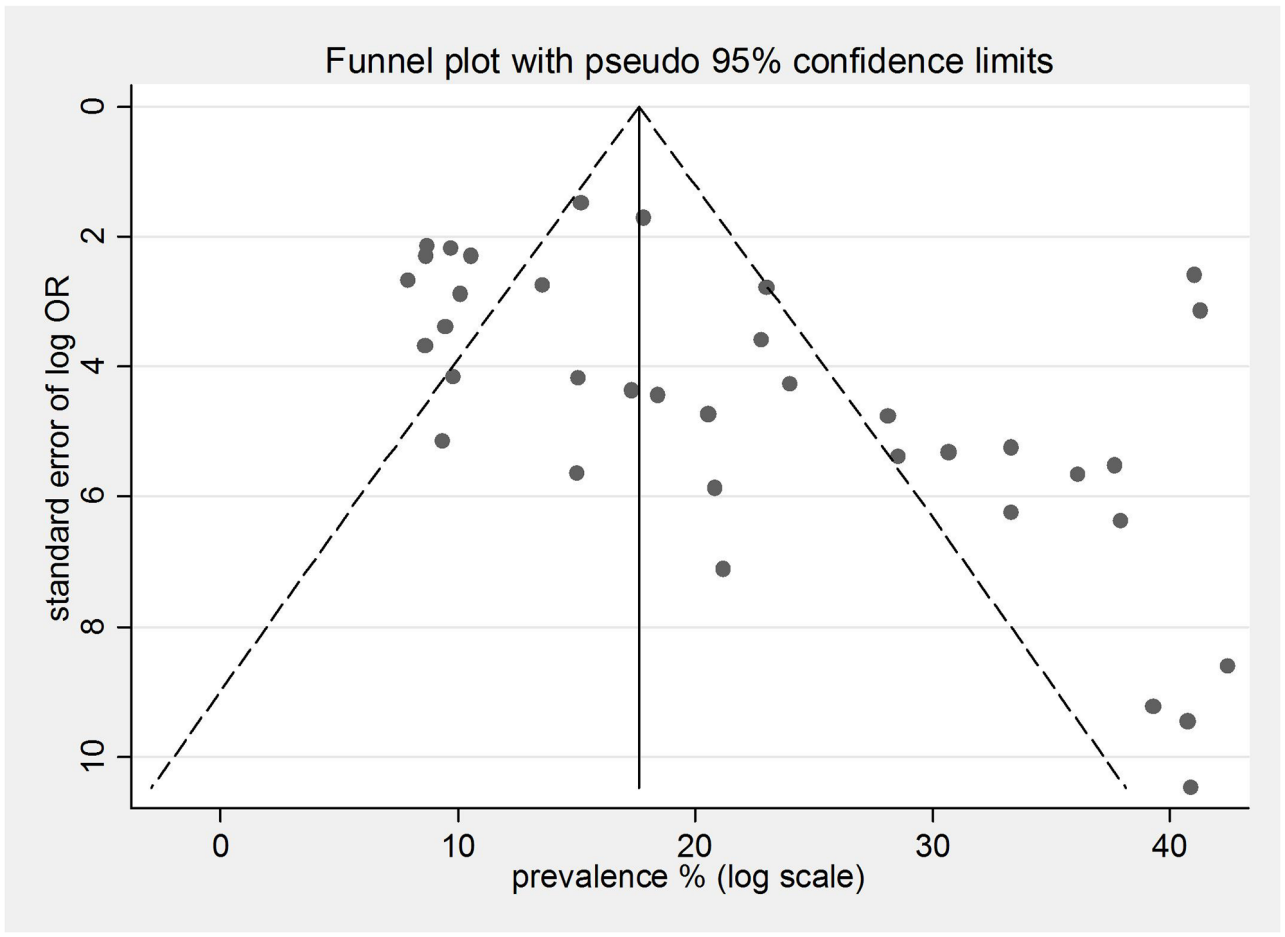
Funnel plot for the included studies that examined small study effects The dashed line represents 95% confidence intervals. Circles represent individual studies.

Publication bias was investigated by funnel plot (Figure 3) and Egger test. Significant publication bias among studies was detected by visual inspection of funnel plot, and there was asymmetrical distribution of the studies indicating publication bias (Egger test P = 0.012).

## DISCUSSION

This systematic review and meta-analysis involved 4518 patients with intracranial tumor from 37 observational studies and demonstrated a high prevalence of depression or depressive symptoms (overall prevalence 21.7%; 95 % CI 18.2%–25.2%). The prevalence is higher than that in normal population, which is up to 4 % of men and 8 % of women [71]. The reason is possibly awareness of disease state and the effect of treatment. But the prevalence is comparably lower than that in patients with diabetes and breast cancer, partly due to its rapid disease progression [72–76]. Brain tumor patients with depression or depressive symptoms are reported to have worse health related quality of life (HRQoL), elevated risk of suicide, more medical complications and worse survival [5, 20, 44, 54, 57]. Unfortunately, only part of patients with depression are properly treated [20]. Thus assessment of depression or depressive symptoms in patients with brain tumor is essential for clinical practitioners to improve prognosis and HRQoL. The role of depression in intracranial tumor patients should be well understood and studied to develop proper management as well.

In explaining the heterogeneity of this meta-analysis, we stratified the groups according to types of depression assessment and found no significant variation in prevalence estimate with clinician-rated depression scales. There were no significant differences between studies in which estimates was made by clinician-rated scales, suggesting that variation between clinical rated tools did not explain the heterogeneity in the symptom prevalence estimates. These results indicated that in the clinical setting, physician based assessing tools are reliable and consistent for depression diagnosis. However, self-rated scales and non-depression-specific scales varied largely in evaluating the estimate prevalence, especially self-rated scales that yielded significantly higher estimates, which could partly explain the heterogeneity [77].

There seems no consensus to define the best standardized scale for assessing the depression or depressive symptoms in brain tumor patients [77]. Therefore, how to accurately assess the prevalence of depression or depressive symptoms and distinguish it from natural reaction is very important [69]. In the study of the association between depression and insulin resistance, Kan et al. divided assessing tools into clinician diagnostic interviews and self-report measures, and observed higher prevalence in the latter group [78]. DSV-IV, HDS and other clinician diagnostic interviews, are validated and consistent in the identification of depression or depressive symptoms. And the patient-reported depression is usually discordant with clinician diagnostic scales [20]. The classification strategy, indeterminate cut-off point and analyzed results indicated the less accuracy and consistence of self-report measures in the diagnosis of depression. However, some self-report measures such as BDI/II, Zung SDS and HADS-D with reasonable cut-off and specific questionnaire could help to screen and assess depression prevalence among brain tumor patients, because they may save time, identify comorbid conditions even with inadequate provider knowledge of the diagnostic criteria, avoid the absence of anonymity and monitor the severity easily [69]. Moreover, non-depression-specific screening methods such as POMS-SF and SF-36 would be better limited into primary epidemiologic screening rather than definite diagnosis, for they recognize distressing emotional symptoms not restricted to depressive symptoms and are associated with low specificity and accuracy [77]. Besides, different depression scales using categorical (yes/no decisions) or dimensional assessment (determined by score or cut-off point) have different estimates of depression, contributing to the heterogeneity [79].

On the other hand, we also investigated correlations between depression prevalence and study characteristics depending on study design, tumor type, sample size, tumor grade, and Newcastle-Ottawa scores. No significant correlation with depression prevalence was found in study design, tumor type and Newcastle-Ottawa scores. Patients with high grade glioma show higher depression prevalence than those with low grade brain tumor. Studies of smaller sample size got an increased depression estimate, suggesting the presence of publication bias. Of the countries patients were recruited, patients from USA had a higher depression prevalence estimate than other countries. This could partly explained by the common use of self-rated assessment tools such as PHQ≥10, BDI ≥10 and CES-D ≥16 [20, 23, 37] and non-depression-specific scales such as POMS-SF [22] and SF-36 [20] in USA.

A secondary analysis during follow-up periods didn’t show an increased prevalence of depression among brain tumor patients after the primary diagnosis. The Relative Increase Ratio in depressive symptoms 1.20, 95% CI (0.91, 1.59), which indicated no remission of depressive symptoms over time. Limited raw data for secondary analysis also indicated the lack of proper monitoring and management of co-morbid depressive symptoms for patients with brain tumor [51].

The study also has some limitations. Firstly, a high heterogeneity in different studies has emerged, although it could be partly explained by different tumor grade, countries and screening methods. Unexamined factors, such as the institutional culture may also play an important role in it [80]. Secondly, the studies included in this meta-analysis didn’t allow understanding the prevalence of depression in brain tumor patients compared with depression prevalence in extracranial tumor patients. It will be better if more stratified cohort studies are conducted to compare different types of brain tumor with health control. More longitudinal studies with constant assessment and management during follow-up periods are necessary to generate more accurate analysis of depression prevalence and prognosis in further studies. Although with few evidence, it remained to be settled down that whether depression symptoms have significant impact on tumor progression and patients’ survival. Diagnosis and treatment of co-morbid depression in brain tumor patients need to be addressed by more studies, and antidepressant therapy or psychotherapeutic intervention for those with co-morbid depression would lead to better life quality and oncology management [19, 20].

## MATERIALS AND METHODS

### Search strategy and inclusion criteria

We searched on PUBMED, PsycINFO and Cochrane library for all peer-reviewed English-language literature from January 1981 through October 2016. The key words used for the database search were: “brain tumor,” OR “intracranial tumors” OR “carcinoma, intracranial,” AND “depression,” OR “depressive symptoms,” OR “depressive disorders,” and the individual corresponding free terms to find more relevant studies (full details of the search strategy are provided in the Supplementary 1). We also searched reviews and meta-analyses to identify studies that may be missed in the former literature searches. Furthermore, all citations in the retrieved articles were obtained and reviewed in full text to search for additional eligible studies [15].

The strategies we used for quality assessment and design protocol is Preferred Reporting Items for Systematic Reviews and Meta-Analyses (PRISMA-P) 2015 guideline [16] (Supplementary 6), which consists of a detailed, well-described checklist for administrative information, introduction, and methods to promote accountability, research integrity, and transparency of the meta-analysis. In addition, we used a modified version of the Newcastle-Ottawa Scale to assess the quality of studies included in systematic reviews and meta-analysis [17]. This scale assessed the quality of studies in the following parts: sample representativeness, sample size, comparability between respondents and non-respondents, outcome of depression diagnosis, and statistical quality (full details in the Supplementary 2). Studies with scores ≥3 points were assessed as low risk of bias, and with scores <3 were in high risk of bias.

All studies published were included if 1) they could be defined as an observational study or a randomized controlled trial which involved patients with brain tumor; 2) All depression screening scales were accepted in the analysis; 3) The diagnosis of brain tumor was according to the guideline of the 2016 World Health Organization (WHO) Classification of Tumors of the Central Nervous System (CNS) in the analysis [18]. We excluded studies without full reports; studies included <20 patients; non–English-language studies; case reports. Only the most informative and/or the recent one will be included if they came from the same authors or the same patient group used in multiple reports.

Two investigators (J. Huang and Chao Zeng) independently performed a systematic review of all identified citations. Papers focusing on selected patients but potentially reporting data about depression were selected for full-text review and checked for eligibility.

### Data extraction and quality assessment of included studies

A standardized data extraction was used by two investigators (J. Huang and Chao Zeng) and checked by the other authors. Any discrepancies were settled by consensus. The following data was abstracted from all included studies: study design, year, country, patients involved, tumor grade, education levels, diagnostic or screening method and prevalence. The demographic and clinical characteristics of the publications included were summarized in Table. When more than one point prevalence estimate of depression would have been recorded in longitudinal studies within the year, the overall period prevalence for the time period was used. It should be also noted that in 10 studies, data were recorded separately for high-grade glioma and low-grade glioma clearly on depression prevalence [4, 6, 7, 14, 19–24].

### Statistical analysis

The prevalence estimates of depression co-morbidity was calculated by random-effects meta-analysis that accounted for between- study heterogeneity [15, 25, 26]. Statistical heterogeneity among studies was assessed using the χ2 test on Cochran’s Q statistic and by calculating I^2^ [27]. I^2^ values of 25%, 50%, and 75% were defined as low, moderate, and high heterogeneity separately [28]. An I^2^ value greater or equal than 50% indicated considerable levels of heterogeneity [27, 28]. We also conducted a sensitivity analysis by serially excluding each study and repeating the meta-analysis to evaluate whether the results were affected statistically significantly by individual studies. Publication bias was evaluated by using funnel plots and the Egger test [29, 30]. Summary estimates of depression for patients with brain tumor were analyzed using Strata software (version 12.1; Stata Corp, College Station, TX). Forest plots were constructed as well. In all analyses, p value <0.05 was considered statistically significant. Where appropriate, if information was available, we compared results from different studies separately based on their characteristics (study design, country, tumor type, sample size, tumor type, tumor grade and diagnostic accuracy) using stratified meta-analysis and subgroup analysis [31, 32].

## SUPPLEMENTARY MATERIALS













## Abbreviations

BDI: Beck Depression Inventory
HADS-D: Depression Subscale of Hospital Anxiety and Depression Scale
DSM-IV: Diagnostic and Statistical Manual of Mental Disorders, 4th. Edition
PHQ-9: Patient Health Questionnaire–9
HDS: Hamilton Rating Scale for Depression
SF-36: 36-Item Short Form Health Survey
BDI-II: Beck Depression Inventory-II
CES-D: Center for Epidemiologic Studies-Depression Scale
BCS: Brief Cope Scale
Zung SDS: Zung Self-rating Depression Scale
POMS-SF: Profiles of Mood States Short Form
GP: General Practitioner (family physician)
SF-36: 36-Item Short Form Health Survey
NR: not applicable
