# Supplementary material for: Association between depression and brain tumor: a systematic review and meta-analysis
Source: Oncotarget. 2017 Aug 3;8(55):94932–43. doi: 10.18632/oncotarget.19843 (PMC5706925; doi:10.18632/oncotarget.19843)
Supplement: Supplementary file 1 [file oncotarget-08-94932-s001.pdf]

Association between depression and brain tumor: a systematic review and meta-analysis

SUPPLEMENTARY MATERIALS

Supplementary 1: Search strategy used in this systematic review and meta-analysis.

See Supplementary File 1

Supplementary 2: Modified Newcastle-Ottawa scoring guide.

See Supplementary File 2

Supplementary 3: Sensitivity analysis.

See Supplementary File 3

Supplementary 4: Meta-analyses of the prevalence of depression or depressive symptoms among brain tumor patients Stratified by study design **(A),** country **(B),** sample size **(C),** tumor type **(D),** assessment type of depression scales **(E)** and Newcastle-Ottawa scores **(F)**.

See Supplementary File 4

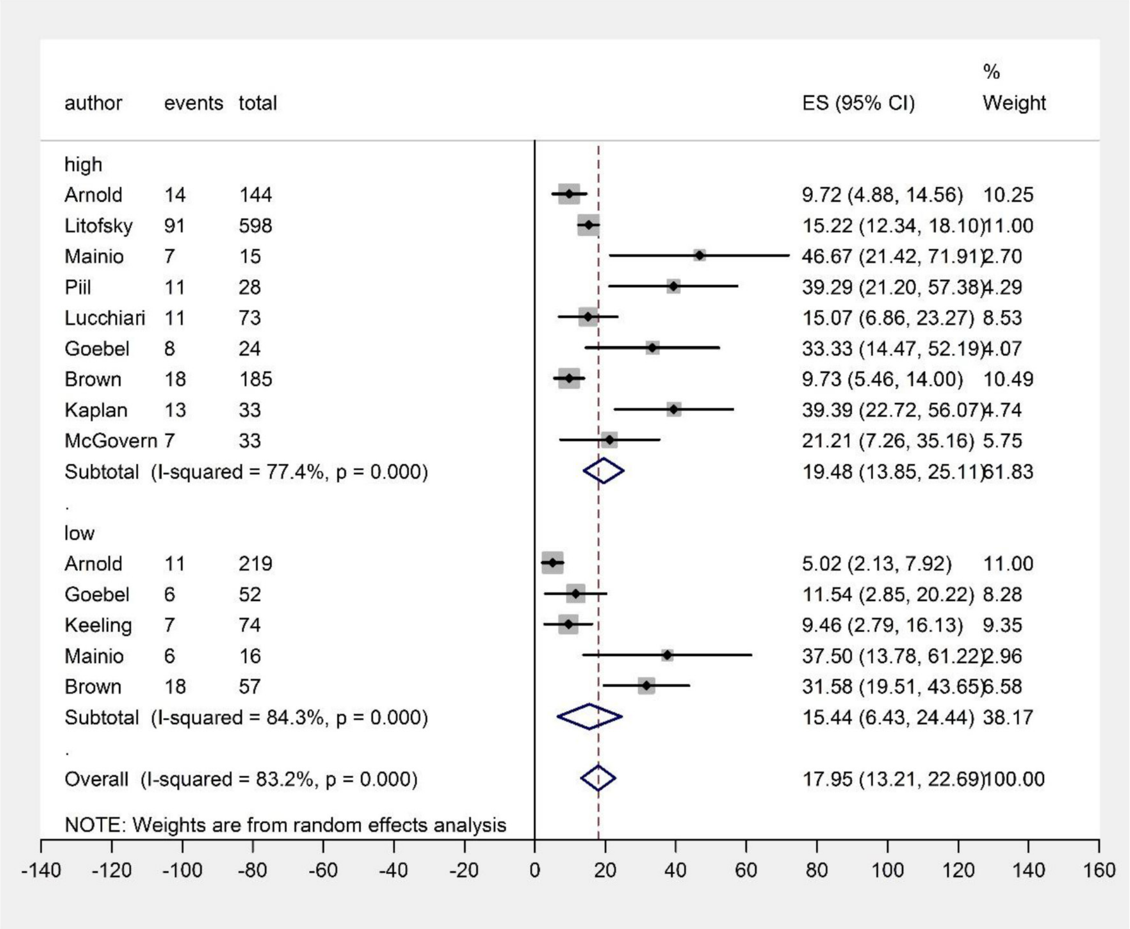

Supplementary 5: Forest plots showing pooled prevalence of depression in high grade brain tumor and low grade brain tumor.

Supplementary 6: PRISMA-P 2015 checklist: recommended items to address in a systematic review protocol*.

See Supplementary File 5
