# Supplementary material for: Association between depression and brain tumor: a systematic review and meta-analysis
Source: Oncotarget. 2017 Aug 3;8(55):94932–43. doi: 10.18632/oncotarget.19843 (PMC5706925; doi:10.18632/oncotarget.19843)
Supplement: Supplementary file 2 [file oncotarget-08-94932-s002.docx]

Supplementary 1. Search strategy used in this systematic review and meta-analysis.

*Depression*

1. Depressed
2. Depression
3. Depression [MeSH]
4. Depressive disorder [MeSH]
5. Depressive disorder, major [MeSH]
6. Major depression
7. Major depressive disorder
8. MDD
9. Sadness
10. Depressive symptoms
11. OR / 1 – 10

*Brain tumor patients*

1. Brain tumor [MeSH]
2. Brain tumor
3. Brain tumor patients
4. Intracranial tumors
5. Intracranial carcinoma
6. Pituitary adenoma
7. Meningioma
8. Glioblastoma
9. Astrocytoma
10. Head and neck
11. OR / 12 – 21

*Study design*

1. Cohort stud*
2. Cohort studies [MeSH]
3. Cross-sectional studies [MeSH]
4. Epidemiologic stud*
5. Epidemiologic studies [MeSH]
6. Incidence
7. Prevalence
8. Longitudinal stud*
9. Meta-analy*
10. Meta-analysis [Publication Type]
11. Observational stud*
12. Prevalence
13. Review [Publication Type]
14. Systematic review
15. OR / 23 – 36

*Combined search*

1. #11 AND #22 AND #37

Abbreviations: MeSH, Medical Subject Heading in MEDLINE.
