# Supplementary material for: Association between depression and brain tumor: a systematic review and meta-analysis
Source: Oncotarget. 2017 Aug 3;8(55):94932–43. doi: 10.18632/oncotarget.19843 (PMC5706925; doi:10.18632/oncotarget.19843)
Supplement: Supplementary file 3 [file oncotarget-08-94932-s003.docx]

Supplementary 2 Modified Newcastle-Ottawa scoring guide.

(1) Representativeness of the sample:

1 point: Tumor types investigated including multiple brain tumor types.

0 points: Tumor types investigated including glioma only.

(2) Sample size:
1 point: Sample size was greater than 100 participants.

0 points: Sample size was less than 100 participants.

(3) Non-respondents:

1 point: Comparability between respondent and non-respondent characteristics was established.

0 points: Comparability between respondent and non-respondent characteristics was not established, or the response rate was unsatisfactory.

(4) Ascertainment of depression:

1 point: Validated depression measurement tool using a validated cutoff score.

0 points: Non-validated measurement tool, or validated measurement tool with non-valid

cut-off score, or 2-item PRIME-MD (scored as such due to its low specificity).

(5) Quality of descriptive statistics reporting:

1 point: Reported descriptive statistics to describe the population (e.g., age, sex) with proper measures of dispersion (e.g., standard deviation, standard error, range).

0 points: Descriptive statistics were not reported, were incomplete, or did not include proper measures of dispersion.

Legend: This scale, the scoring of which ranges from 0 to 5, assesses quality in several domains: sample representativeness and size, comparability between respondents and non-respondents, ascertainment of depressive symptoms, and statistical quality. Studies were judged to be of low risk of bias (≥3 points) or high risk of bias (<3 points).

| Author | Year | Representativeness | Size | Comparability | Outcome | Statistics | Total |
| --- | --- | --- | --- | --- | --- | --- | --- |
| anderson | 1999 | 1 | 0 | 0 | 1 | 1 | 3 |
| Andrewes | 2012 | 1 | 0 | 0 | 1 | 1 | 3 |
| ANGELO | 2008 | 1 | 0 | 0 | 1 | 1 | 3 |
| Armstrong | 2002 | 0 | 0 | 0 | 1 | 1 | 3 |
| Arnold | 2008 | 1 | 1 | 0 | 1 | 1 | 3 |
| Brown | 2006 | 0 | 1 | 0 | 1 | 1 | 3 |
| Bunevicius | 2013 | 1 | 1 | 0 | 1 | 1 | 4 |
| CHANG | 2003 | 0 | 1 | 0 | 0 | 0 | 1 |
| Davies | 1996 | 1 | 0 | 0 | 0 | 1 | 2 |
| Edelstein | 2015 | 0 | 0 | 0 | 1 | 1 | 2 |
| Giovagnoli | 1996 | 1 | 1 | 0 | 1 | 1 | 3 |
| Goebel | 2010 | 1 | 1 | 0 | 1 | 1 | 4 |
| Goebel | 2012 | 1 | 1 | 0 | 1 | 1 | 4 |
| Goebel | 2011 | 1 | 1 | 0 | 1 | 1 | 4 |
| Goebel | 2012 | 1 | 1 | 0 | 1 | 1 | 4 |
| Grant | 2010 | 0 | 0 | 0 | 1 | 0 | 1 |
| Hickmann | 2016 | 1 | 0 | 0 | 1 | 1 | 3 |
| Janda | 2007 | 1 | 0 | 0 | 1 | 1 | 3 |
| Jenkins | 2015 | 1 | 0 | 0 | 1 | 0 | 2 |
| Kaplan | 2000 | 1 | 0 | 0 | 1 | 0 | 2 |
| Keeling | 2012 | 1 | 0 | 0 | 1 | 1 | 3 |
| Kilbride | 2011 | 1 | 0 | 0 | 1 | 1 | 3 |
| Leistner | 2015 | 0 | 1 | 0 | 1 | 1 | 3 |
| Litofsky | 2004 | 0 | 1 | 0 | 1 | 1 | 3 |
| Lucchiari | 2014 | 0 | 0 | 0 | 1 | 1 | 2 |
| Mainio | 2006 | 0 | 0 | 0 | 1 | 1 | 2 |
| McGovern | 2003 | 1 | 0 | 0 | 0 | 0 | 1 |
| Pelletier | 2002 | 1 | 0 | 0 | 1 | 1 | 3 |
| Piil | 2015 | 0 | 0 | 0 | 1 | 1 | 2 |
| Pringle | 1999 | 1 | 1 | 0 | 1 | 1 | 4 |
| Rahman | 2015 | 1 | 0 | 0 | 1 | 1 | 3 |
| Rooney | 2009 | 0 | 1 | 0 | 0 | 1 | 2 |
| Rooney | 2011 | 0 | 1 | 0 | 1 | 1 | 3 |
| Santini | 2012 | 1 | 0 | 0 | 0 | 1 | 2 |
| Vossen | 2014 | 0 | 1 | 0 | 1 | 1 | 3 |
| WELLISCH | 2002 | 1 | 0 | 0 | 1 | 1 | 3 |
| Wenz | 2015 | 0 | 0 | 0 | 0 | 1 | 1 |
