# Supplementary material for: Association between depression and brain tumor: a systematic review and meta-analysis
Source: Oncotarget. 2017 Aug 3;8(55):94932–43. doi: 10.18632/oncotarget.19843 (PMC5706925; doi:10.18632/oncotarget.19843)
Supplement: Supplementary file 4 [file oncotarget-08-94932-s004.docx]

Supplementary 3. Sensitivity analysis.

|  | Author | Year | Prevalence (%) | lower 95% CI | Upper 95% CI | Tau^2^ | I^2^ |
| --- | --- | --- | --- | --- | --- | --- | --- |
| Omitting | Wenz | 2015 | 21.0 | 17.5 | 24.5 | 0.00909 | 89.10% |
| Omitting | Santini | 2012 | 20.3 | 16.9 | 23.7 | 0.00891 | 89.10% |
| Omitting | Armstrong | 2002 | 20.3 | 16.9 | 23.8 | 0.00891 | 89.10% |
| Omitting | Hickmann | 2016 | 20.6 | 17.2 | 24.0 | 0.00901 | 89.00% |
| Omitting | Kaplan | 2000 | 20.3 | 16.9 | 23.7 | 0.00888 | 89.10% |
| Omitting | Leistner | 2015 | 19.8 | 16.6 | 23.0 | 0.00738 | 86.80% |
| Omitting | Mainio | 2006 | 20.6 | 17.2 | 24.0 | 0.00901 | 89.00% |
| Omitting | Pelletier | 2002 | 20.2 | 16.8 | 23.6 | 0.00878 | 89.00% |
| Omitting | Edelstein | 2015 | 20.6 | 17.2 | 24.1 | 0.00916 | 89.30% |
| Omitting | Rooney | 2011 | 20.6 | 17.2 | 24.0 | 0.00901 | 89.00% |
| Omitting | WELLISCH | 2002 | 20.6 | 17.2 | 24.0 | 0.00900 | 89.00% |
| Omitting | Rooney | 2009 | 20.6 | 17.1 | 24.0 | 0.00912 | 89.20% |
| Omitting | Andrewes | 2012 | 20.6 | 17.2 | 24.0 | 0.00901 | 89.00% |
| Omitting | Bunevicius | 2013 | 20.6 | 17.1 | 24.1 | 0.00927 | 89.20% |
| Omitting | Goebel | 2010 | 21.0 | 17.6 | 24.5 | 0.00914 | 88.80% |
| Omitting | Goebel | 2012 | 21.0 | 17.6 | 24.5 | 0.00915 | 88.80% |
| Omitting | Goebel | 2011 | 21.0 | 17.5 | 24.5 | 0.00933 | 89.00% |
| Omitting | Goebel | 2012 | 20.9 | 17.4 | 24.4 | 0.00920 | 89.20% |
| Omitting | Grant | 2010 | 20.6 | 17.2 | 24.0 | 0.00912 | 89.30% |
| Omitting | Janda | 2007 | 20.7 | 17.2 | 24.2 | 0.00920 | 89.30% |
| Omitting | Keeling | 2012 | 20.6 | 17.2 | 24.0 | 0.00901 | 89.00% |
| Omitting | Kilbride | 2011 | 20.9 | 17.5 | 24.4 | 0.00912 | 89.20% |
| Omitting | Lucchiari | 2014 | 20.8 | 17.3 | 24.3 | 0.00920 | 89.30% |
| Omitting | Piil | 2015 | 20.3 | 16.9 | 23.7 | 0.00890 | 89.10% |
| Omitting | Pringle | 1999 | 21.0 | 17.5 | 24.5 | 0.00920 | 89.10% |
| Omitting | Rahman | 2015 | 20.3 | 16.8 | 23.7 | 0.00885 | 89.00% |
| Omitting | Vossen | 2014 | 20.6 | 17.1 | 24.1 | 0.00919 | 89.20% |
| Omitting | anderson | 1999 | 20.8 | 17.3 | 24.2 | 0.00913 | 89.30% |
| Omitting | McGovern | 2003 | 20.6 | 17.2 | 24.1 | 0.00909 | 89.30% |
| Omitting | Jenkins | 2015 | 20.2 | 16.8 | 23.6 | 0.00881 | 89.00% |
| Omitting | Davies | 1996 | 19.9 | 16.5 | 23.4 | 0.00874 | 89.10% |
| Omitting | Arnold | 2008 | 19.7 | 16.6 | 22.7 | 0.00657 | 85.30% |
| Omitting | CHANG | 2003 | 20.8 | 17.2 | 24.4 | 0.01004 | 89.30% |
| Omitting | Brown | 2006 | 21.0 | 17.5 | 24.5 | 0.00927 | 88.90% |
| Omitting | Litofsky | 2004 | 20.9 | 17.3 | 24.6 | 0.01028 | 89.30% |
| Omitting | ANGELO | 2008 | 21.0 | 17.5 | 24.5 | 0.00914 | 89.20% |
| Omitting | Giovagnoli | 1996 | 21.1 | 17.6 | 24.5 | 0.00906 | 88.90% |
|  | Pooled estimate |  | 20.6 | 17.2 | 24.0 | 0.00901 | 89.00% |
