# Supplementary material for: Association between depression and brain tumor: a systematic review and meta-analysis
Source: Oncotarget. 2017 Aug 3;8(55):94932–43. doi: 10.18632/oncotarget.19843 (PMC5706925; doi:10.18632/oncotarget.19843)

Supplementary 4. Meta-analyses of the Prevalence of Depression or Depressive Symptoms Among brain tumor patients Stratified by study design (A), country (B), sample size (C), tumor type (D), assessment type of depression scales (E) and Newcastle-Ottawa scores (F).


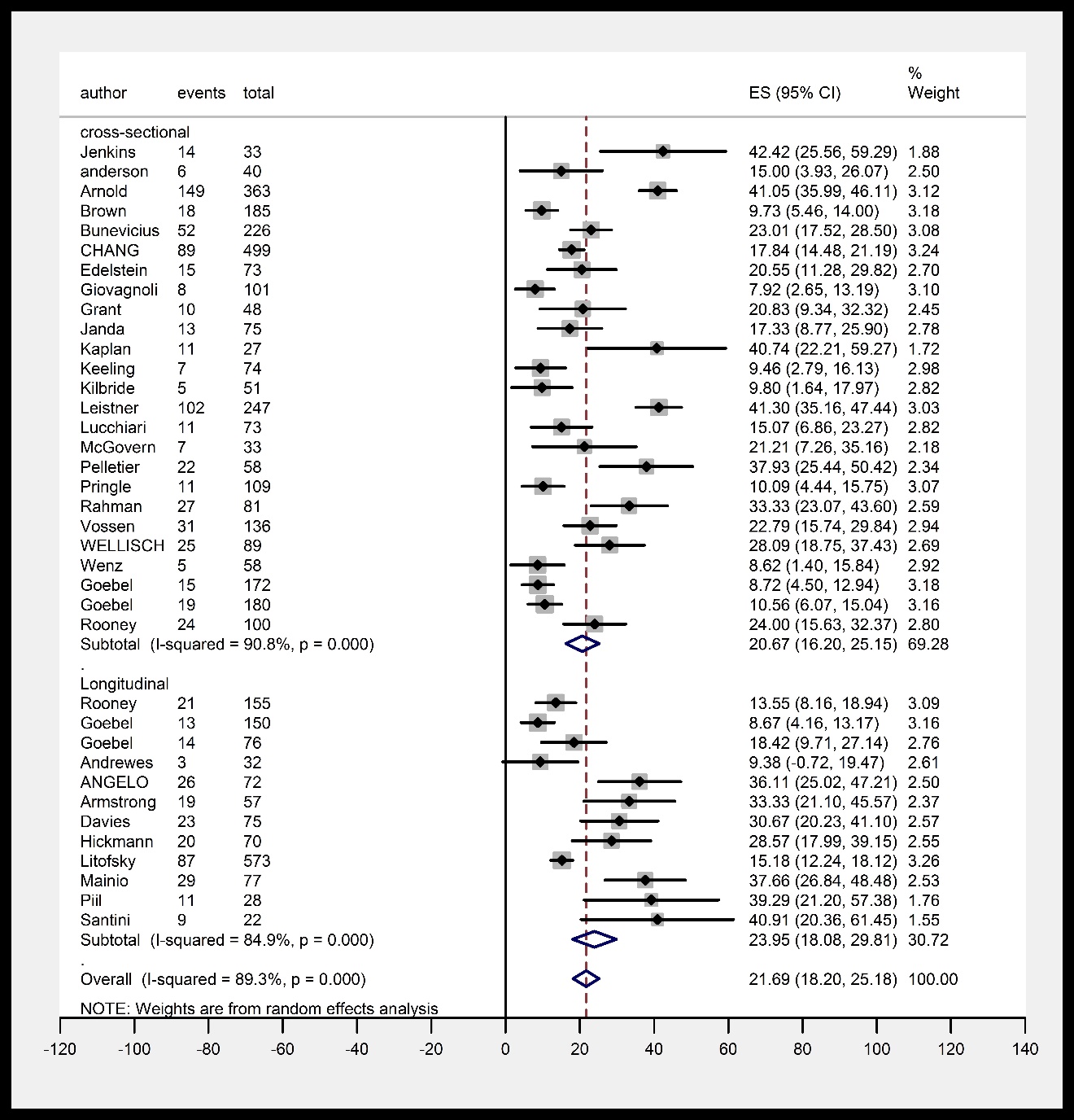


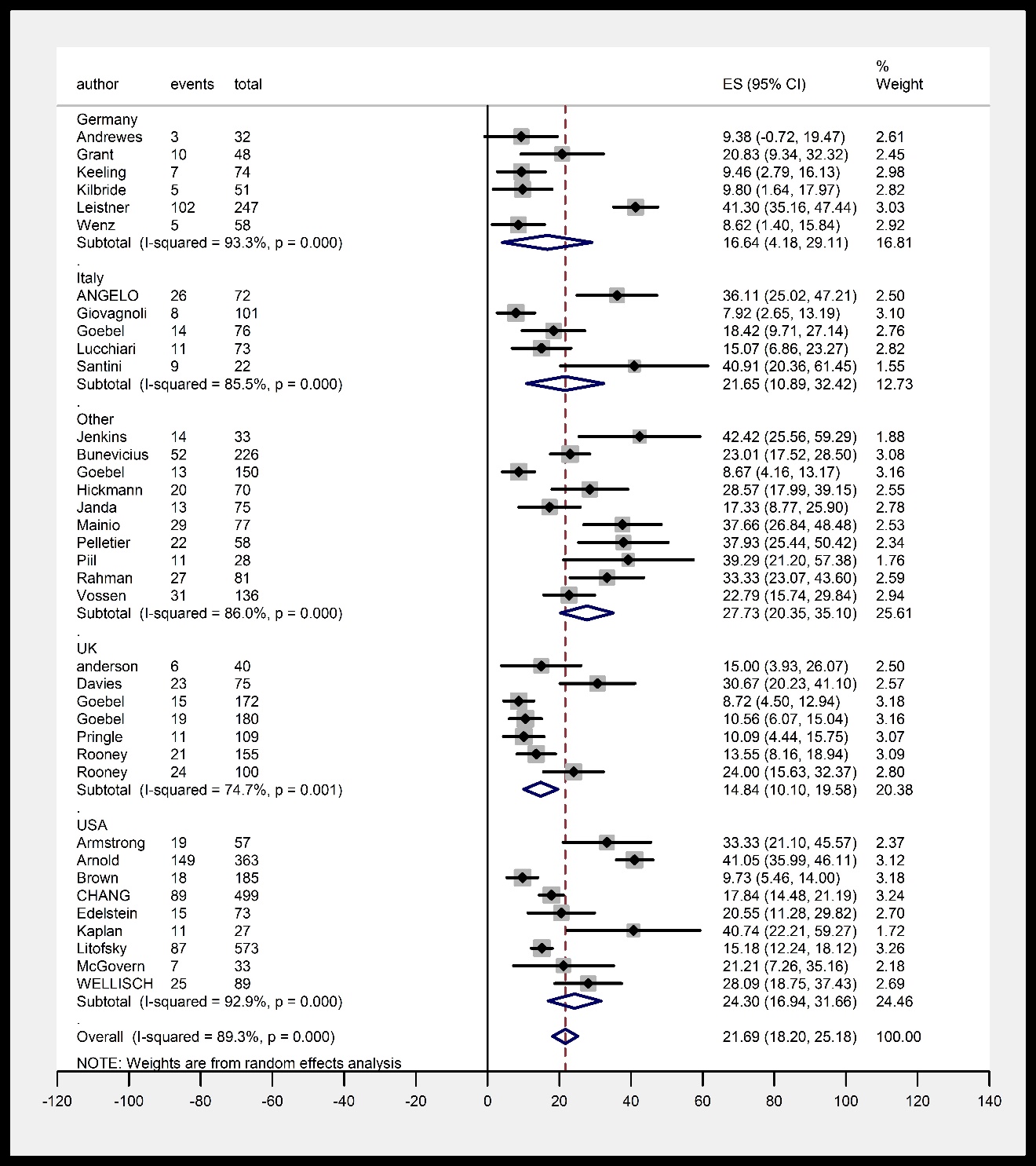


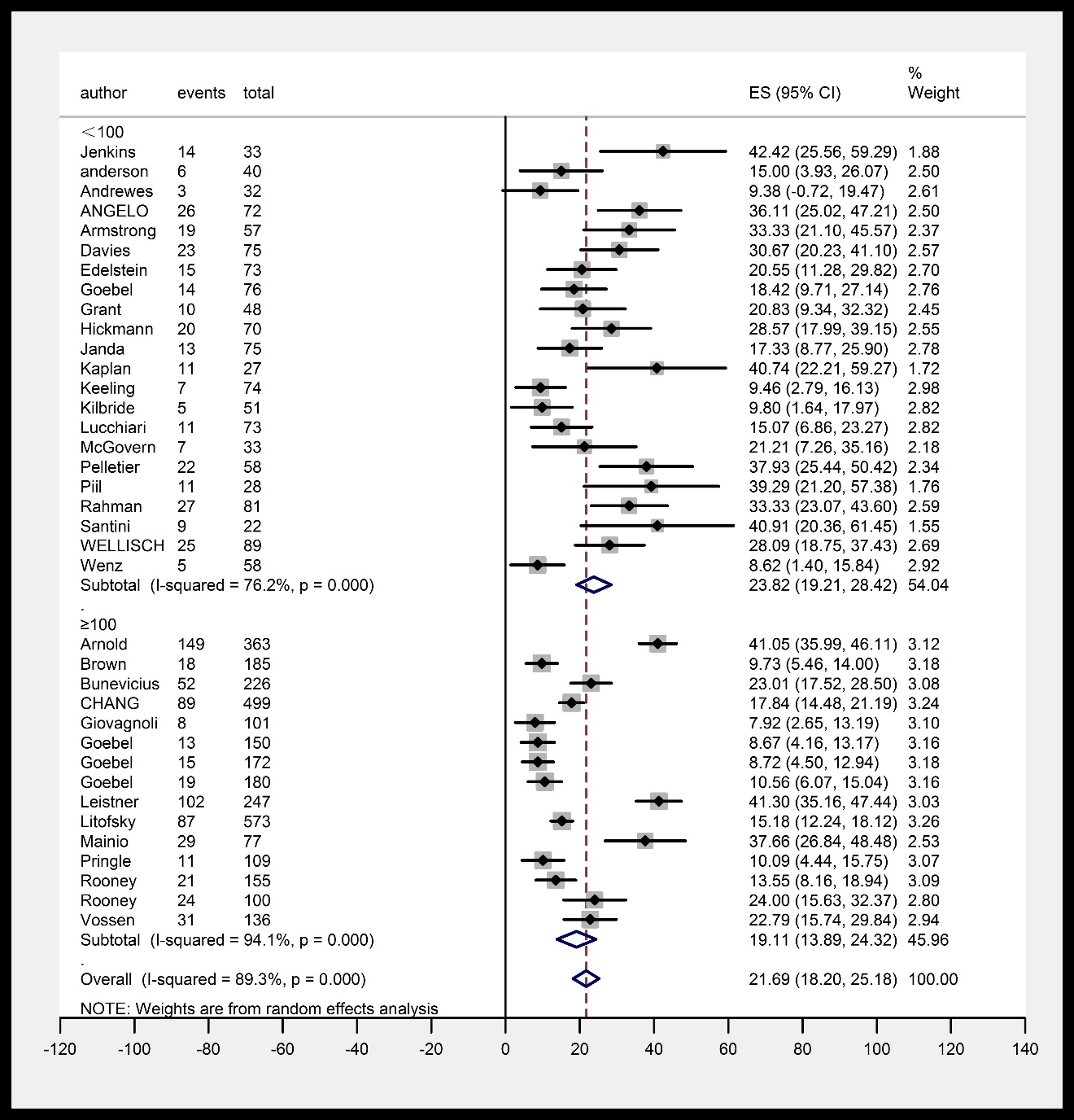


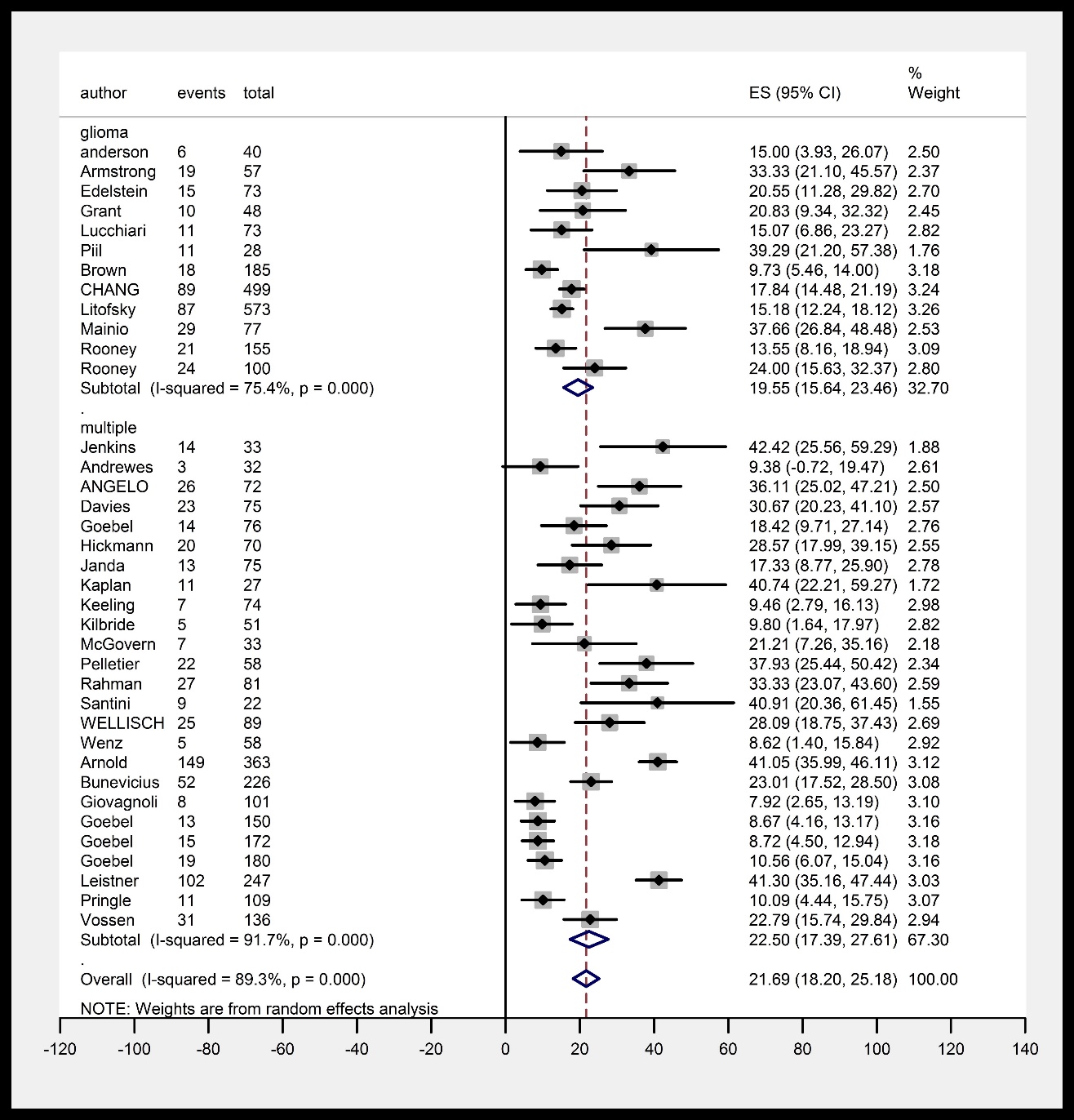


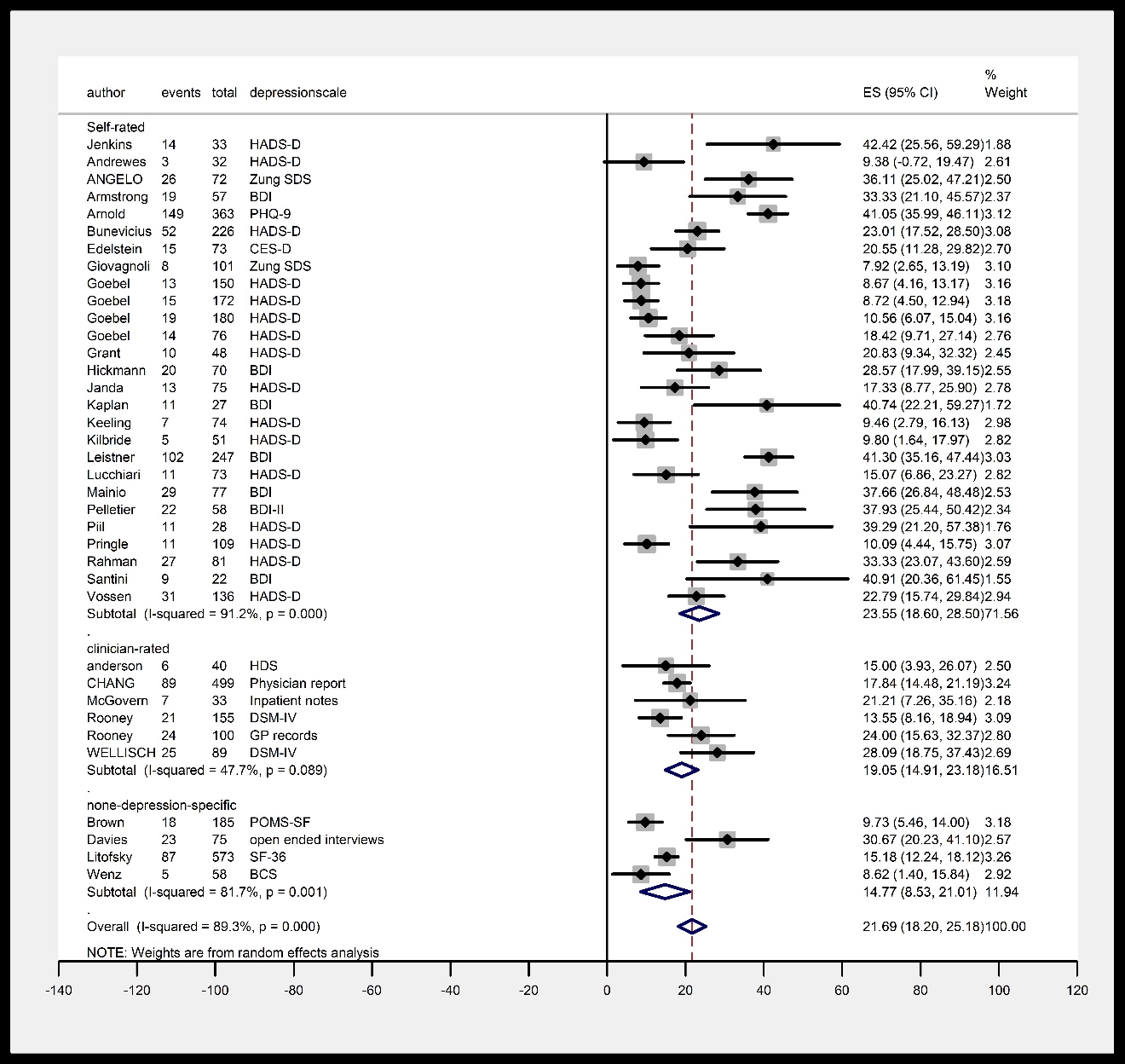

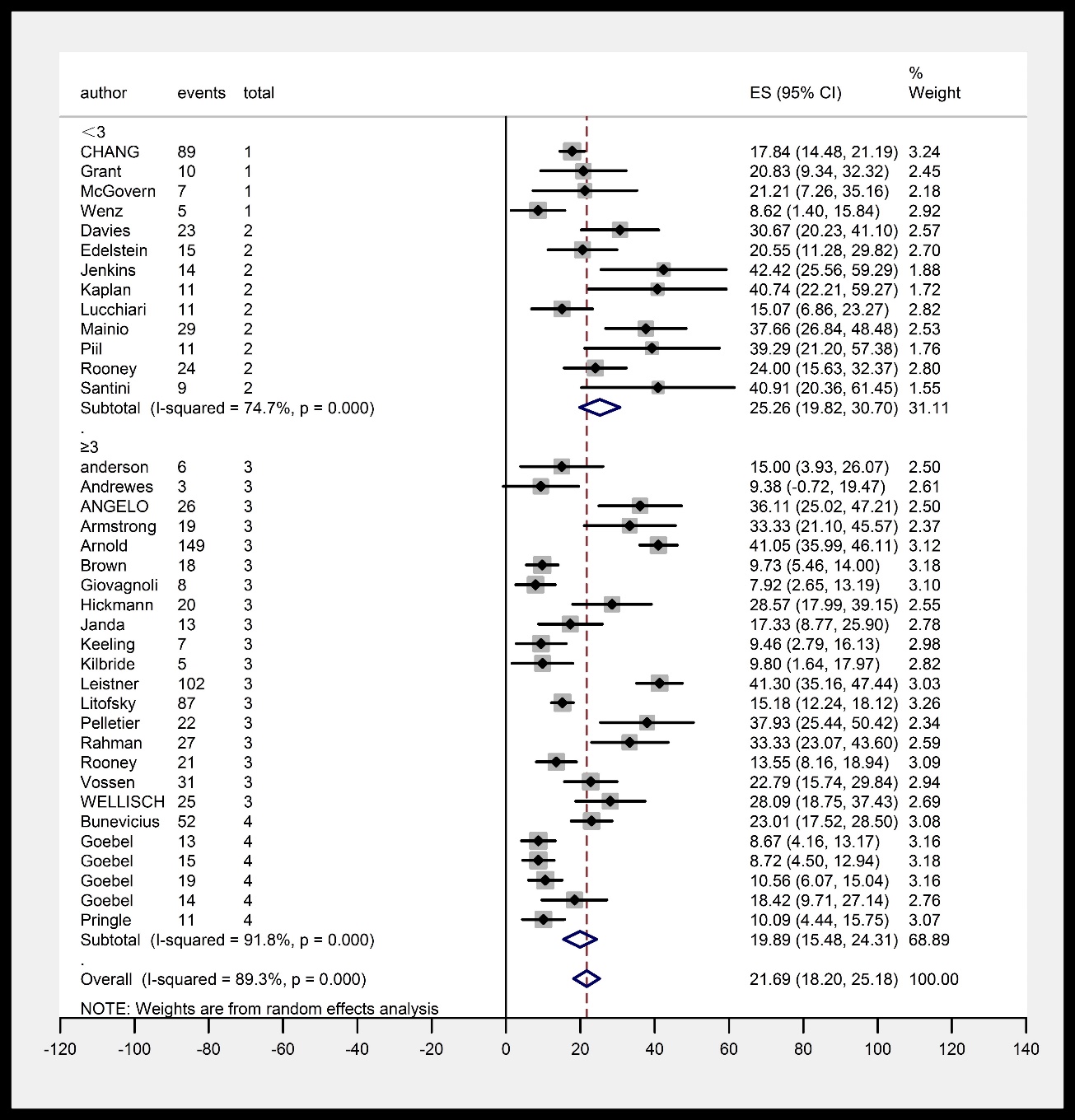

Supplement: Supplementary file 5 [file oncotarget-08-94932-s005.docx]
